# Activity‐based funding for safety and quality: A policy discussion of issues and directions for nursing‐focused health services outcomes research

**DOI:** 10.1111/ijn.12775

**Published:** 2019-08-15

**Authors:** Liza Heslop

**Affiliations:** ^1^ Institute for Health and Sport Victoria University Melbourne Victoria Australia

**Keywords:** activity‐based funding, health policy, hospital‐acquired complications, nursing, safety and quality

## Abstract

**Aims:**

A discussion of the implications and opportunities arising from the Commonwealth of Australia health care reform agenda; linking pricing with quality, with particular reference to directions for nursing‐focused health services outcomes research directed to improve the safety and quality of health care practices.

**Background:**

National activity‐based funding in Australia is a policy‐focused development. As the relationship between cost and quality becomes apparent, the role of clinicians and their contribution to high quality care has become a pressing issue for leadership, teaching, and research.

**Design:**

Discussion paper

**Data Sources:**

This paper is based on seven years' experience as a member of a Commonwealth of Australia statutory committee—the Clinical Advisory Committee of the Independent Hospital Pricing Authority—and is supported by relevant literature and theory.

**Implications for Nursing:**

To date, unravelling the linkage, especially causal relationships, between direct care nursing and patient safety outcomes has not been well established. New activity‐based funding data elements developed for national implementation in Australia provide accessible and meaningful standardised data for measurement of never events, hospital‐acquired complications, and preventable readmissions.

## SUMMARY STATEMENT

What is already known about this topic?

- The advancement of research directed towards finding causal associations attributing nursing interventions to patient outcomes has been constrained by, amongst other things, methodological challenges
- The attribution of nursing care interventions to specific patient‐related outcomes is difficult to isolate
- Investigating how nursing care interventions contribute to safety and quality health care outcomes is often referred to as the “black box” of nursing‐focused health services outcomes research

What this paper adds?

- Research into the impact of nursing interventions on patient outcomes, such as hospital‐acquired complications, remains immature
- Activity‐based funding data provide safety and quality measures relevant to nursing‐focused health services outcomes research
- Building clinical‐decision support, based on the Australian Commission for Safety and Quality in Healthcare hospital‐acquired complication outcome measures, may assist nurses engage with quality improvement as nurses are likely to act on data relevant to their practice

The implications of this paper:

- The Australian Commission for Safety and Quality in Healthcare hospital‐acquired complication outcome measures have enhanced data specifications, useful to support development of nursing‐focused health services outcomes research
- The potential for benchmarking of hospital‐acquired complications is high at least in Australia and in other countries that apply activity‐based funding models linked to ICD‐10‐AM codes

## 1 INTRODUCTION

Adopted by more than 30 countries, Activity Based Funding (ABF) has become the international model for funding hospital‐based care and is referred to by many terms, such as case‐mix funding or payment by results (Baxter et al., 2015). ABF is based on services provided to patients and the efficient price of providing those services with adjustments for patient populations served. As a robust technology, ABF has created different opportunities for clinicians, operational managers, and modern research agendas. Enhancements to ABF data that classify errors in health care practices provide opportunities that are highly relevant to contemporary nursing research and practice. Errors leading to adverse events pose high risks to patients and are costly from a human, economic, and social viewpoint. In an era of health care budgetary austerity, it has become abundantly apparent that a reduction in the rate of adverse events such as hospital‐acquired complications (HACs) could potentially produce productivity savings, as well as direct benefits to patients.

Over several years of detailed work, the Clinical Advisory Committee assisted the Australian Commission for Quality and Safety in Healthcare (ACSQHC) and the Independent Hospital Pricing Authority (IHPA) to identify options for incorporating safety and quality into the pricing and funding of public hospital services alongside partners that included clinicians, jurisdictional representatives, and other key stakeholders. This discussion paper is guided by the question: how can ABF contribute to improve the safety and quality of nursing care in the hospital setting? Whilst this paper discusses the use of the ABF platform in Australia, many of the points and issues raised are internationally relevant. I explain the genesis of ABF policy in Australia, the imperatives that recent pricing for safety and quality bring to patient safety in the health care setting, and the implications arising from this policy for leveraging relevant nursing‐focused health services outcomes research. There are opportunities arising from the availability of enhanced ABF data, where nursing‐focused outcomes research could improve known gaps concerning the operationalisation of patient safety measurement variables and better understand the interrelationships between nursing interventions and patient safety outcomes, as well as reduce the incidence of adverse events, particularly HACs.

The context of this discussion paper lies within the Australian public health care system, though the issues and points I raise here will resonate with many international health care jurisdictions. It is important to note that ABF models, in general, do have common objectives, but are at different stages of data classification, development, and implementation. One cannot speak about an ABF model per se as each differs from country to country on many levels. For example, in European hospitals today, ABF is the most common mechanism for reimbursing hospitals, though most classifications there, unlike the Australian ABF model, do not discriminate between diagnoses present on admission (comorbidities) and those occurring during the hospital stay (complications) (Or, 2014).

## 2 DISCUSSION

### 2.1 The development of ABF in Australia

After ABF was introduced in the late 1990s in Victoria, Australia, initial commentary in health care management and policy literature was sceptical of the reform objectives of ABF. Concerns were raised regarding the ability of ABF to provide a fair basis for funding hospitals, achieve overall budget reduction, and improve efficiency of public hospitals (Braithwaite, Hindle, Phelan, & Hanson, 1998). In 1998, the Auditor‐General of Victoria, Ches Baragwanath, investigated the impact of ABF in Victoria, reporting that achievement of high‐level efficiency gains was met through case‐mix funding (Baragwanath, 1998). The report raised concerns, however, that the narrow policy focusing on efficiency gains had impacted negatively on aspects of the quality of patient care. More recently, Australian health care policy officials claim that enhancements to how public hospital funding is determined are proving effective—not only in terms of efficiency, but also because ABF enables providers and clinicians to intervene in health service improvement proactively, including aspects of safety and quality (see Downie, 2017).

In Australia, implementation of the Commonwealth acute health reform strategy commenced in July 2012. From 2015 to 2016 onwards, the Australian ABF model serviced all admitted programmes: admitted acute, nonadmitted services (aggregated data), nonadmitted services (patient‐level data), emergency (aggregated data), emergency (patient‐level data), admitted subacute and nonacute, and mental health care. Implicit to the reform was the national adoption of ABF to fundamentally drive, amongst other benefits, allocative efficiency and incentives for hospitals at the operational level. Health care institutions capture patient data from clinical records for the purposes of classification and ABF (Refer to Table 1 for information on the Australian ABF model process of classification.) Detailed data set specifications are readily accessible to jurisdictions. See, for example, the current version of metadata items for the data set specifications for the Admitted Patient Care National Minimum Data Set 2017‐2018 (Ihpa.gov.au, 2018a). Thus, as part of the mandatory inpatient Commonwealth activity‐based reporting systems, a large amount of data is collected for each inpatient episode. The data elements have use beyond ABF and have been effective for clinical epidemiological studies, research into the quality of health care and patient safety, utilisation review, and for providing demographic, financial, cost, and length of stay information. With clinician review of ABF data, length of stay decreases, more efficient models of care have emerged, and considerable efforts have been directed to minimise complications of care (Bohlouli, Jackson, Tonelli, Hemmelgarn, & Klarenbach, 2017; Larg, Moss, & Spurrier, 2018). Even so, there remain considerable gaps between the policy volume of work produced and its use by clinicians, administrators, and planners (McCrow, 2016).

**Table 1:** Process for classifying acute admitted episodes of care

Classifying or “coding” involves the translation of documentation from the patient's clinical record into alphanumeric codes within ICD‐10‐AM and the Australian Classification of Health Interventions (ACHI)
Following patient discharge, clinical coders review patients' medical records, abstract recorded clinical documentation, and assign codes for the principal diagnosis, additional diagnoses, and procedures performed
Guidelines for coding are provided in the Australian Coding Standards
Following the assignment of ICD‐10‐AM and ACHI codes, episodes of care are assigned to a DRG in the Australian refined diagnosis‐related groups (AR‐DRG) classification.
The process of assigning patient episodes to a DRG is complex and completed using software that contains AR‐DRG algorithms (referred to as “the Grouper”).
IHPA has continued to contract ICD‐10‐AM/ACHI/ACS development to the Australian Consortium for Classification Development (ACCD) for the Eleventh Edition, whilst the development of AR‐DRG V10.0 is being undertaken by IHPA.
ACCD has finalised ICD‐10‐AM/ACHI/ACS Eleventh Edition for implementation from 1 July 2019. AR‐DRG V10.0 has also been finalised.
AR‐DRGs are used in all public and private hospitals in Australia, and the classifications are updated every 2 years to ensure that they are fit for purpose and remain clinically current.

### 2.2 Hospital‐acquired complications and ABF policy

The use of Australian ABF data has assisted with estimates of hospital‐acquired diagnoses as well as providing compelling evidence about the economic benefits for improving their cost impact (Bail et al., 2015; Kjellberg et al., 2017; Pearse, Mazevska, & Jackson, 2015). For instance, Pearse et al. (2015) reported that in 2011/12, 2% of Australian public hospital separations had a HAC (Australian Commission on Safety and Quality in Health Care, 2016). Further, a study conducted for the ACSQHC estimated that a hospital‐acquired diagnosis increased the average cost of a hospital admission by $9200, with an incremental impact on length of stay of 5.3 days (Australian Commission on Safety and Quality in Health Care, 2013)

Recent work by the ACSQHC and IHPA has determined a list of high priority adverse events known as HACs (Table 2). The list was achieved following lengthy and comprehensive clinician‐driven processes of consultation, data modelling, literature reviews, and testing within public and private hospitals. The HAC list has been developed in an evidenced‐based way to distinguish those complications which are preventable and that have the greatest patient impact (severity), clinical priority, and health service impact. Although the HAC list includes HAC05 “unplanned intensive care unit admission,” this currently cannot be measured because the information required to identify an unplanned intensive care unit admission is not collected in the current dataset specification and thus cannot be identified (refer to Ihpa.gov.au, 2018a). An outcome of the HAC policy initiative has been to prioritise the type of errors that health service organisations should address.

**Table 2:** Australian Commission for Safety and Quality in Healthcare: hospital‐acquired complications (Ihpa.gov.au, 2018b)

**1** Pressure injury
**2** Falls resulting in fracture or other intracranial injury
**3** Health care‐associated infection
**4** Surgical complications requiring unplanned return to theatre
**5** Unplanned intensive care unit admission
**6** Respiratory complications
**7** Venous thromboembolism
**8** Renal failure
**9** Gastrointestinal bleeding
**10** Medication complications
**11** Delirium
**12** Persistent incontinence
**13** Malnutrition
**14** Cardiac complications
**15** Third and fourth‐degree perineal laceration during delivery
**16** Neonatal birth trauma

For the purposes of this discussion paper, I refer to “hospital‐acquired complications” using the abbreviation (HACs), as determined by the IHPA and defined by the national list for which clinical risk mitigation strategies may reduce (but not necessarily eliminate) the risk of that complication occurring. The HACs can be identified in ABF data, as the HACs are specifically flagged as a code named “Condition Onset Flag = 1”. The HACs have not previously been systematically addressed in Australia. In the United States, there has been a decade of intense regulatory focus on the prevention of HACs (Wald, 2017). Internationally, solutions to minimise these forms of harm have been researched (Spetz et al., 2013, Boyle, Bergquist‐Beringer, & Cramer, 2017; Lyren et al., 2017), including understanding what can be done to prevent, better understand, and address health care‐associated infections (Kaba, Baumann, Kolotylo, & Akhtar‐Danesh, 2017).

A recent development in the Australian ABF model has been to incorporate quality signals. A risk‐adjusted model with technical specifications (Version 1.0 July 2017) for each HAC forms the basis for funding adjustment and was under consultation on the IHPA Pricing Framework for 2018‐2019 (Ihpa.gov.au, 2018b). In 2018‐2019, IHPA introduced a HAC Adjustment into the Pricing Framework so that funding varies according to the patient's risk of developing a HAC during the episode of care. The risk adjustment model pricing mechanisms allow for variations in a range of patient complexity factors such as age, palliative care status, and care type so that hospitals that treat more high‐risk patients are not financially disadvantaged compared with hospitals that treat fewer such patients.

The IHPA is currently consulting on pricing and funding approaches for avoidable hospital readmissions where risk‐adjusted funding approaches have been used extensively in other countries such as Denmark, Germany, England, and the United States. Readmissions are of concern. For example, figures on the costs of the Australian State of Victoria readmissions show that of 16 045 readmissions with a primary diagnosis of a “complication of surgical or medical care” amounted to $70.6 million per year on public expenditure on these cases (McNair, Jackson, & Borovnicar, 2010).

### 2.3 Contemporary challenge of nursing‐focused health services outcomes research

The linking of health pricing with quality is a reflective policy development that may help improve sustainable change on harmful consequences arising during the episode of care; nevertheless, a response to use of ABF data to drive improvement efforts requires collaborative efforts and research. Research into the safety and quality of health care is often underpinned by Donabedian's structure‐process‐outcome (SPO) model of health care improvement (Ayanian & Markel, 2016). This model has helped nursing‐focused health services outcomes researchers structure complex relationships between structural (S), process (P), and outcomes (O) measures. Studies by Gardner, Gardner, and O'Connell (2014), Pitkäaho, Partanen, Miettinen, and Vehviläinen‐Julkunen (2016), and Tvedt, Sjetne, Helgeland, and Bukholm (2012) demonstrate this in more detail. A growing body of successful research has been directed towards understanding how characteristics of the nurse workforce (a structural variable on the Donabedian model) are associated with patient‐related safety and quality outcomes—an outcome variable on the Donabedian model (Bachnick, Ausserhofer, Baernholdt, & Simon, 2018; Kim & Bae, 2018; Patrician et al., 2017; Smith, Morin, Wallace, & Lake, 2018; Tourangeau, Giovannetti, Tu, & Wood, 2016).

For some time, Linda Aiken, Clarke, Sloane, Sochalski, and Silber (2002) have been instrumental in discovering relationships between nurse staffing characteristics and patient outcomes, more recently showing associations between reduced registered or professional nurse levels and mortality (Aiken et al., 2017). Other important nursing workforce research contributions showing associations between nurse staff levels and patient outcomes include studies on “missed care” (see, for example, Griffiths, et al., 2018; Jones, Hamilton, & Murry, 2015). The general conclusion of contributions to this significant body of research, largely from the United States and Canada, is that care and patient outcomes are substantially better when there is a higher proportion of bachelor‐degree prepared nurses employed (Needleman, 2015). Even so, a systematic review by Stalpers, de Brouwer, Kaljouw, and Schuurmans (2015) investigating associations between characteristics of the nurse work environment and five nurse‐sensitive patient outcomes in hospitals found evidence to support such associations, although results remain equivocal as clear conclusions were often missing from studies, including poor sample sizes that lack sufficient power to detect clinical relevance. Thus, for this category of research whilst showing the importance of nursing resource deployment to support the safety and quality of health care provision, and alerting policy officials at all levels about the need for appropriate professional nurse staffing levels to manage patient‐related safety and quality outcomes, nursing researchers have argued that more attention be directed towards peering into the “black box”—that being the development of empirical understandings regarding the impact of direct care nursing interventions on patient outcomes, and not limited to structural variables of nurse characteristics and nurse staffing (Heslop & Lu, 2014; Kim, Lyder, McNeese‐Smith, Leach, & Needleman, 2015). One of the greatest challenges for contemporary nursing‐focused health services outcomes research is to attribute the effect of nursing interventions on patient‐related safety and quality outcomes. Considerable complexity of, and variation within, the measurement of nursing‐sensitive patient safety and quality outcomes requires researchers to undertake extensive and costly validation of dependent outcome safety and quality measures. Many measures can be best described as proxy measures. That is, researchers identify single or multiple indicators that they and others from various local or international contexts agree can effectively be used as widely defined measures of optimal practice. This represents something of a compromise and hinders knowledge synthesis from previous research about the nursing contribution to safety and quality. The downside, then, for nursing knowledge development concerns constraints on knowledge accumulation—so important for development of practically oriented, midrange theory of the nurse practice environment.

### 2.4 Implications for nursing‐focused health services outcomes research using ABF data

Underpinned by well‐developed data standards and associated specifications, the HAC data platform makes available quality and safety outcome measurement. Infections, pressure injuries, and malnutrition are HACs highly relevant to direct care input by nurses and have been deemed nurse sensitive (Aydin, Donaldson, Stotts, Fridman, & Brown, 2015; D'Amour, Dubois, Tchouaket, Clarke, & Blais, 2014). For instance, hospital‐acquired malnutrition arising from deterioration in nutritional status occurs in almost 70% of inpatients leading to increased morbidity, increased length of stay, and a culminative effect on a range of indicators such as infection, wound healing, and delirium (Kirkland & Shaughnessy, 2017). Eide, Halvorsen, and Almendingen (2015) found that undernourished elderly are not identified and treated properly and that improvements to nutritional care practices on hospital wards were needed. Hospital‐acquired malnutrition is a HAC where nurses will be able to lead and partner with teams to design strategies to reduce harm arising from deteriorating nutritional status.

As mentioned, how nursing care processes are potentially involved in the correction of these HACs is not well understood, and this is where future research opportunities lie for nursing‐focused outcomes research. First, the specifications for the HACs list have been updated to include the 10th edition of the ICD‐10‐AM. These specifications have been available to monitor HACs since 1 July 2017. The specifications include a changelog that outlines differences between the 9th and 10th editions of the codes. Secondly, the ACSQHC and the IHPA have developed Excel and SAS tools (also known as groupers) that can be used by hospitals, health services, and system managers to identify and monitor HACs using their data. The SAS grouper requires specific software and expertise. Thirdly, ACSQHC and IHPA have developed an animation called The Medical Record and Data‐Driven Healthcare. Available on YouTube, it is intended to raise awareness of data uses generated from the medical record and encourages improvements in clinical documentation. Plans are also in place for development of a suite of educational tools aimed at improving clinical documentation, benchmarking, and service planning with one application recently released to assist frontline clinical staff record care processes using accurate terminology that meets requirements drawn from ICD‐10‐AM and the Australian Coding Standards. The app WRITEitRIGHT is a quick reference tool to support clinical documentation in Australian hospitals and is free to download from the App Store or Play Store. The app prompts when a user should move from general to specific terminology with a directory of clinical terms and diagnoses.

In addition to these resources, a national benchmarking portal hosted by the New South Wales, Ministry of Health (Ihpa.gov.au., 2018c) allows users to compare cost and activity (for example: Diagnostic‐Related Group [DRG]; Principal diagnosis; Principal procedure) from Australian hospitals. Use of the portal gives the ability to analyse system wide safety and quality matters and compare hospital differences in activity, cost and efficiency, and the incidence of HACs in a cost‐effective way. The HAC data have high methodological strength and offer a viable alternative to the development of nurse registries of pressure injury data in Australia where known deficits exist (Heslop, 2015).

#### 2.4.1 Health service methodologies

A health service methodology known to enhance effective use of ABF data is clinical utilisation review (CUR). CUR can be supported by data mining approaches to analyse patient‐level discharge data (McCrow, 2016). ABF data can be reviewed and mined at many levels: internal peer, service type, facility, local health network, and jurisdictional and national levels. Health services CUR analyses can provide real‐time evidence‐based, clinical‐decision support. In addition, CUR strategies provide for identification of opportunities for improvement in service quality (through better support of unwarranted clinical variation), service availability (through better use of existing services, where there is clinical indication), or a reduction in service cost. The process of CUR does require input from relevant experts or clinical analysts in order to guide the framework for analysis and to identify interactions that are not merely of statistical interest, but also of potential operational value. Further, data mining or manual intervention may be required to direct the analysis process. For example, when developing models that identify factors influencing length of stay, it may be advisable to exclude day patients from the analysis.

### 2.5 Limitations

Quality enhancement continues to be a complex process that requires organisational commitment, adequate infrastructure and resources, change champions, and a personal commitment to quality care (Baxter et al., 2015). Fundamental to quality enhancement, as pointed out in compelling evidence from nursing‐focused health outcomes research, would be appropriate levels of qualified nursing staff with expertise in the use and application of evidence in practice. ABF is not the panacea to support quality monitoring and reporting but has appropriately incorporated quality dimensions as an object of its policy. A wide range of tools are already available to clinicians for quality improvement purposes such as computerised discharge abstracts, data from clinical support systems, round table type data, and cost data. These and other clinical‐decision support tools may be used in conjunction with the ABF data.

Concerns about elements of ABF including the potential for data manipulation and gaming have been raised (de Jong, 2018; Neby, Lægreid, Mattei, & Feiler, 2015). But there are processes and incentives in place in Australia to ensure that there is no gaming. Australian Coding Standards provide rules which enforce what can be coded (Shepheard, 2017). States and territories of Australia are required to have audit and independent oversight mechanisms in place to ensure that the coding standards are adhered to. Supported by transparent governance and a focus on development and improving the consistency of coded data, ABF is underpinned by advanced health technologies in Australia that have been shaped over time. The IHPA monitors coded data they receive, and any evidence of wide scale gaming would be brought to the attention of the relevant state. If gaming or rule bending opportunities become created within the Australian public health, such behaviours would be a significant challenge to the public ethos of limiting costly and preventable complications.

Another common criticism of ABF models concerns their failure to accurately measure resource use. For example, differences in nursing resource use appear not to be accurately captured in case‐mix groupings (Heslop, 2012). Functional levels of mobility and self‐care are important concepts of nursing care captured routinely in nursing and allied health practice. There could be a value to adding “functioning” information into ABF models which is largely uncaptured (Hopfe et al., 2015). When assessing for risk of pressure injury, for example, nurses tend to focus on patient factors of care dependency and self‐care, these factors being established as important to nurses' perception of patient risk (Balzer et al., 2014). Nurse decision making for risk management of pressure injuries has elements external to the use of standardise pressure injury risk assessment tools. As ABF models are neither static nor concrete but are rather evolving systems and technologies, it would be important for policy officials to consider evidence provided by Hopfe et al. (2015) for, in particular, functioning levels of classification may provide a future for ABF models servicing chronic disease management such as the emerging ABF models for nonadmitted services.

Although I have suggested an operational solution for the potential use and transfer of rich ABF data that are well‐developed and validated to better quantify nurse‐related quality of care outcome measures, there remain complex methodological challenges associated with applying this evidence to nursing‐focused health services outcomes research. Griffiths et al. (2016) provide a useful summary of methodological improvements needed for cross‐sectional studies that, for example, explore relationships between nurse staffing levels and quality of care, and provide also a checklist to aid future cross‐sectional study development.

## 3 CONCLUSION

The progress of systematic measurement of safety and quality outcomes sensitive to nursing practice are essential components for a scientifically grounded profession. Nursing‐focused health services outcomes research often report measures of adverse events that lack correspondence and consistency. Sixteen high priority safety and quality indicators, known as HACs in Australia, provide standardised data with defining attributes and empirical referents based upon definitive, coded, clinical documentation from the patient's clinical record.

Use of the ABF classification scheme will help overcome methodological shortfalls associated with definitions and operationalization of patient safety and quality variables. With the use and application of HACs, opportunities are likely to arise for improved data synthesis across Australian hospitals and potentially with other countries that apply ABF models linked to the international classification coding scheme ICD‐10‐AM. Nursing‐focused health services outcomes research has strengthened linkages between the nursing contribution and adverse events, although much more needs to be done. Such research, as it continues, will better enable nurses, hospital administrators, and policy and decision makers to more fully understand how nursing interventions impact upon the prevention and management of HACs. With better use of ABF data, nurses will be able to lead multidisciplinary initiatives to support the early identification and prevention of adverse events and take up leading roles in reducing hospital readmissions.

Finally, because sorting out the differential contributions that direct care nursing interventions make to safety and quality outcome measures remains immature—in the sense that cause‐and‐effect relationships need improving—it remains unclear to me at this stage if it is worth investing, or even feasible and practical, to continue down this line of inquiry—that being the research focus of attributing, or indeed isolating, specific nursing care interventions associated with the prevention or minimisation of adverse events. Perhaps, it may be more fruitful to consider multidisciplinary approaches—such as the effect of bundled multidisciplinary care pathways on adverse events—as it is well‐known that complex interventions contain several interactive components. This approach would not clarify the nurses' unique contribution to health care but the desired product of nursing and why nursing matters. Additionally, as nursing‐focused health services outcome researchers attempt to progress the evidence base of the nursing discipline, the call now is to orient this area of importance with a firmer focus on the impact of interventions or process of care.

## CONFLICT OF INTEREST STATEMENT

The authors declare no conflict of interest.

## DISCLAIMER

The views expressed in this publication do not necessarily reflect the views of authorities of the Australian Government. Responsibility for the information and views expressed in this discussion paper lies entirely with the author.
